# Corticomuscular Coherence and Motor Control Adaptations after Isometric Maximal Strength Training

**DOI:** 10.3390/brainsci11020254

**Published:** 2021-02-18

**Authors:** Dimitri Elie, Franck Barbier, Ghassan Ido, Sylvain Cremoux

**Affiliations:** 1 UMR CNRS 8201—LAMIH, Université Polytechnique des Hauts de France, F-59313 Valenciennes, France; dimitri.elie@uphf.fr (D.E.); franck.barbier@uphf.fr (F.B.); 2 Centre Hospitalier de Saint-Amant-les-Eaux, 59230 Saint-Amant-les-Eaux, France; gido@chsa.fr; 3 Centre de Recherche Cerveau et Cognition, UPS, Université de Toulouse, 31052 Toulouse, France; 4 Centre de Recherche Cerveau et Cognition, CNRS UMR 5549, 31052 Toulouse, France

**Keywords:** plantarflexion, training performances, EEG, EMG

## Abstract

Strength training (ST) induces corticomuscular adaptations leading to enhanced strength. ST alters the agonist and antagonist muscle activations, which changes the motor control, i.e., force production stability and accuracy. This study evaluated the alteration of corticomuscular communication and motor control through the quantification of corticomuscular coherence (CMC) and absolute (AE) and variable error (VE) of the force production throughout a 3 week Maximal Strength Training (MST) intervention specifically designed to strengthen ankle plantarflexion (PF). Evaluation sessions with electroencephalography, electromyography, and torque recordings were conducted pre-training, 1 week after the training initiation, then post-training. Training effect was evaluated over the maximal voluntary isometric contractions (MVIC), the submaximal torque production, AE and VE, muscle activation, and CMC changes during submaximal contractions at 20% of the initial and daily MVIC. MVIC increased significantly throughout the training completion. For submaximal contractions, agonist muscle activation decreased over time only for the initial torque level while antagonist muscle activation, AE, and VE decreased over time for each torque level. CMC remained unaltered by the MST. Our results revealed that neurophysiological adaptations are noticeable as soon as 1 week post-training. However, CMC remained unaltered by MST, suggesting that central motor adaptations may take longer to be translated into CMC alteration.

## 1. Introduction

The ability to reach and maintain a certain amount of force is crucial in daily life activities, e.g., to hold a shopping bag or keep the foot onto the brake pedal. The stability of the strength production is highly driven by the concurrent activation of agonist and antagonist muscles [1,2], respectively acting in and against the direction of the net joint torque production. Numerous authors highlighted that training procedures alter the concurrent activation of agonist and antagonist muscles [3,4], which changes the motor coordination, i.e., the stability and the accuracy of the force production [5]. Some of these adaptations appear to directly occur through the communication between the brain and the muscles [6,7]. The communication between the brain and the muscles can be quantified with the corticomuscular coherence (CMC), that is, the spectral relationship between M1 electroencephalographic (EEG) and electromyographic (EMG) oscillatory activities. To date, the dynamic of CMC change during strength training remains mostly unknown. This study investigates corticomuscular coherence changes during 3 weeks of strength training. 

Numerous studies have shown that the motor cortex (M1) is highly involved in controlling voluntary muscle contractions [8,9]. During muscle contraction, the M1 activity recorded by EEG is in synchrony with both agonist and antagonist EMG signals [7,8,10,11,12,13]. Most studies report significant CMC in alpha (α, 8–13 Hz) and beta (ß, 13–31 Hz) frequency bands [8,14,15]. There is a strong consensus that ß-band CMC reflects descending information from the M1 to the muscle contributing to the movement execution [8]. The literature is sparser regarding the α-band CMC. It has been supposed to reflect ascending or feedback interactions [8,16]. While a study that investigated short-term visuomotor training revealed significant increase of the CMC-ß magnitude [17], another study showed lower CMC-ß magnitude with agonist muscles in strength-trained (ST) experts and ballet dancers in comparison to the untrained control group [11]. This dynamic adaptation of CMC-ß magnitude with training was interpreted as, at first, an intensive cortical solicitation of the physiological processes involved in motor control to accurately perform the motor task, then, secondly, an efficiency of motor control once the training intervention is over. These results were later corroborated by Dal Maso et al. (2017) [7]. The authors also evidenced higher CMC-ß magnitude with the antagonist muscle in the ß-band in ST experts in comparison to endurance-trained athletes. The authors hypothesized that the higher CMC-ß magnitude revealed in ST experts could reflect a higher supraspinal involvement in the control of the antagonist muscle. These results show that ST performed over several years modulates the communication between M1 and agonist and antagonist muscles. However, the neural adaptations are supposed to have the first role in motor training [18]. Indeed, it seems that only four weeks of hand function motor training combined with electrical stimulations could already alter the CMC in the ß-band [19]. Another study evidenced that a few days of motor training could also enhance the accuracy and the stability of the torque production [20] and altered the CMC in the α-band, suggesting a possible relationship between the CMC and motor coordination. Therefore, it is likely that the quantification of CMC could help in monitoring the neural adaptations induced by the training intervention.

For a similar force level, the antagonist muscles are less activated in ST experts than endurance-trained participants [4,6]. These observations suggest that the training specificity leads to different antagonist contributions regarding torque production. The stability and the accuracy of the force production are also improved after strength training [5,21]. Numerous strength training procedures and modalities have been investigated in the literature. Most studies revealed that few repetitions of high load intensity exercise lead to higher strength improvement [22,23,24,25]. This type of training is known as maximal strength training (MST) and consists of 4 or 5 sets of 4 to 5 movement repetitions performed at nearly maximal intensity (e.g., ~90 ± 5% of the 1-RM), three times a week [26,27]. The MST appears promising to induce sharp neurophysiological adaptations to develop or recover strength production as soon as 3 weeks of training. Interestingly, the ST procedure has been shown to improve motor coordination by enhancing the stability and the precision of the force production during submaximal contractions [5,21,28,29]. The motor coordination can be evaluated by the variability and the accuracy of the force production [5,21,28,29]. A recent study investigated the variability and the accuracy of the torque production, and the CMC-ß magnitude with agonist and antagonist muscles according to the force production phase, i.e., increasing, holding or decreasing force phase [10]. The authors hypothesized that an increase in the CMC-ß magnitude with agonist and antagonist muscles leads to a better motor coordination, quantified by a reduction of motor production variability. All of these results suggest that the CMC-ß magnitude could concomitantly be modulated by the ST procedure and the motor coordination improvement induced by the ST procedure.

This study evaluated the motor adaptations and their underlying neural adaptations induced by a 3 week MST specifically designed to strengthen the plantarflexion (PF) in novice participants. Motor adaptations were quantified through regular evaluation of the maximal torque production and the variable error (VE) and absolute error (AE) during submaximal contractions to detect any motor control changes. Due to the MST procedure’s specificity, we expected a sharp increase in PF maximal torque production. We hypothesized that these motor adaptions would be associated with better motor coordination during submaximal contractions. The cortical and muscle adaptations were quantified through changes of the CMC magnitude and the activation level of both agonist (Triceps Surae) and antagonist (Tibialis Anterior) muscles. Although the literature suggest that main muscle adaptations should occur with antagonist muscle [4,6,30], we hypothesized an overall decrease of both agonist and antagonist muscle activation and alteration in ß-band CMC after the MST. 

## 2. Materials and Methods

### 2.1. Subjects

Thirteen healthy men (27.6 ± 6.7 years; 1.77 ± 0.04 m; 76.0 ± 9.9 kg) participated in this experiment. All but one of the participants were right-footed, as assessed by inventory of foot preference [31]. Participants had no known neurological disorders or lower-limb musculoskeletal injuries. Two participants were excluded from the analysis because they failed to comply with the experimental or training procedure; their performances were not included in this study. Before taking part in the experiment, participants were fully informed about the investigation and gave their written informed consent to participate according to the Declaration of Helsinki. The local Ethics Committee of the Université Polytechnique Hauts-de-France (UPHF) approved the present study (ethic approval code: 2017-A044).

### 2.2. Materials

A custom-designed calibrated dynamometer [32] was used to record the isometric net joint around the ankle of the dominant leg at 2048 Hz during the evaluation and training sessions. Participants were seated on a weight bench. Their trunk and pelvis were firmly strapped onto the back of the bench. The dominant leg was placed in the orthosis of the dynamometer and firmly strapped at the level of the thigh, the calf, the ankle, and the metatarsophalangeal joints of the foot to maintain the position of the lower limb throughout the evaluation or training session.

Both EMG and EEG signals were recorded at 2048 Hz using a Refa amplifier (TMSi, Oldenzaal, The Netherlands) during the evaluation sessions only. Following skin preparation, EMG surface electrodes were placed on the tibialis anterior (TA), gastrocnemius lateralis (GL), soleus (SOL), and gastrocnemius medialis (GM), according to the SENIAM recommendations [33]. Electrode placements were marked off to carefully replace electrodes to the same position throughout the longitudinal experiment. A 64-channel EEG was placed according to the 10–20 positioning system to record EEG activity from Fp1, F3, Fz, F4, C3, Cz, C4, P3, Pz, and P4 electrodes [34] to cover the cortical areas associated with movement preparation and execution of the PF. The reference electrode was placed on the head of the left ulna. All recording systems were synchronized offline with a digital trigger.

### 2.3. Experimental Setup

**Figure 1:**
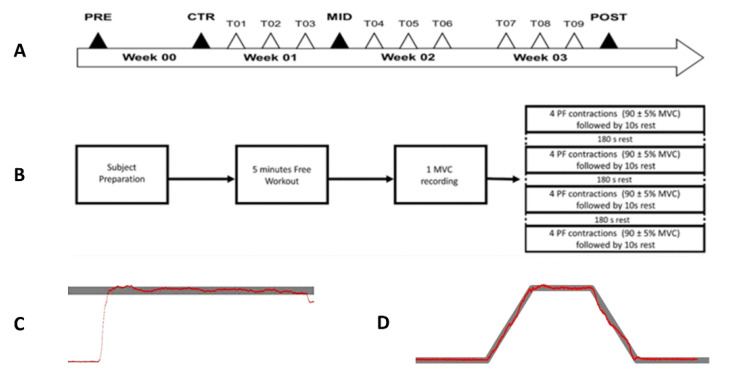
(**A**) Experimental design timeline including the experimental sessions (PRE, CTR, MID, and POST; black triangles) and training sessions (T; white triangles). (**B**) Progress of one training session. (**C**) Typical representation of the net torque feedback (N.m; red line) and target line (grey bar) as a function of time (s) during the training session. (**D**) Typical representation of the net torque feedback (N.m; red line) as a function of time (s) during one trial of the evaluation session.

A 4 week longitudinal experiment was designed to strengthen (training sessions) and evaluate (evaluation sessions) the torque production during plantarflexion contraction. The experimental workflow is depicted in Figure 1A. The first week consisted of initial overall ankle torque evaluation in plantarflexion and dorsiflexion. The following 3-week period was dedicated to Maximal Voluntary Isometric Contractions (MVIC)-based MST intervention performed in plantarflexion 3 times a week. During each training session, the torque production and the training performances were monitored. The second and fourth weeks included evaluation sessions to follow the evolution of ankle torque production, muscle activities, and the CMC magnitude. 

### 2.4. Training Sessions

Participants were trained three times per week during three weeks, totaling nine training sessions that lasted 25 min. Participants performed a free 5 min workout, including running, jumping, PF, and dorsiflexion (DF) exercises before the training session. One training session included one MVIC and four training sets (Figure 1B). The MVIC was only performed in PF. An auditory cue indicated the beginning and the end of the MVIC. Participants were intensively encouraged to develop the maximal torque as fast as possible [10,32] and maintain it throughout the duration of the MVIC. A visual feedback of the torque was given in real time on a screen located 1 m in front of the participant. The maximal mean of a 2 s sliding window ran over the torque produced during the MVIC was defined as the MVIC reference for the following training sets. The first out of four sets began after a 3 min rest. One set included 4 PF isometric contractions at a 90 ± 5% MVIC. On average, the participant maintained 87.23 ± 1.28% of the MVIC during 3.22 ± 0.33 s across all training sessions (see Supplementary Materials for detail). The torque feedback was presented on a screen as a function of time together with the threshold limits (Figure 1C). Through muscle contraction, participants had to move the torque feedback within the threshold limits as fast as possible and maintain the contraction until feedback disappeared. Each contraction lasted 7 s and was followed by a 10 s rest. Between each training set, a 3 min rest period was granted. Two training characteristics were computed offline to objectively quantify the training’s accuracy (see Supplementary Materials for details). 

### 2.5. Evaluation Sessions

Four evaluation sessions were performed throughout the experiment. Two evaluation sessions (PRE and CTR) were conducted a week apart before the first training session. A mid-term evaluation session (MID) was realized after one week of training, and the final evaluation session (POST) was performed at the end of the training. A 48 h rest was respected between each evaluation session and the preceding or following training session.

Each evaluation session consisted of preliminary to three 5 s MVIC in PF and DF as detailed in the training session. The contraction type (PF, DF) was presented randomly. A 2 min rest period was respected between each MVIC. Participants then performed three sets of 20 isometric PFs. These contractions were equitably distributed over two torque levels to be reached and randomly assigned to 20% of the MVIC reached during the PRE session and 20% of the daily MVIC in PF. PRE evaluation contractions were all 20% daily-MVIC. 

Each trial lasted 20 s and consisted of 5 s rest, 3 s linear increasing force phase, 4 s holding force phase, 3 s linear decreasing force phase to return at the resting state, and a 5 s rest. The net ankle torque and the target line to follow were presented in real time (Figure 1D). The thickness of the target line was set as ±0.5% of the associated MVIC. One training set was granted before each evaluation. During the experimental procedure, participants were asked to communicate only in case of discomfort and blink only during the rest period to minimize artifacts in EEG signals. A 2 min rest was observed in between each set. 

### 2.6. Data Processing

Data analysis was performed using Matlab (R2015b Mathworks Inc., Natick, MA, USA). 

#### 2.6.1. Preprocessing 

Prior to any analysis, EEG signals were 3–100-Hz bandpass filtered using 4th-order, zero-lag butterworth filter and visually scrutinized [10]. None of the interest electrodes were identified as bad channels during the experimental procedure and the average-reference was performed using EEGLAB toolbox [35]. Each trial was then inspected and trials containing muscle and/or blink artifacts were excluded from subsequent analysis. On average, 3.91 ± 1.30 trials were removed across all torque levels and evaluations. 

#### 2.6.2. Net Ankle Torque Processing 

The net torque was 10 Hz low-pass filtered using 4th-order, zero-lag butterworth filter [36]. Both PF and DF MVICs were defined as the highest average of a 2 s sliding window ran over the contraction period. The best MVIC out of the three trials was selected for the subsequent analyses. Modulation of MVICs was respectively expressed as a percentage of PRE evaluation. 

For submaximal contractions, the net torque was quantified over a 3 s period of interest centered on the trial (8.5 s to 11.5 s) independently for each torque level during PRE, CTR, MID, and POST evaluations. The percentage torque was quantified separately for each torque level over the period of interest by normalizing the net torque by the daily MVIC. The absolute error (AE) and variable error (VE) of the torque were quantified independently for each torque level over the period of interest and normalized by their respective MVIC. The VE (1) and AE (2) were respectively quantified using the following equations:

(1) $$VE=\frac{\sqrt{\Sigma {\left(x_{i}-\bar{x}\right)}^{2}/n}}{MVIC}$$

(2) $$AE=\frac{\left(\Sigma \;\left|x_{i}-T\right|\right)/n}{MVIC}$$

where $x_{i}$ represents the *i*th torque value, $\bar{x}$, the average torque production over the period of interest, n, the number of values within the period of interest, and, T, the target torque production to be reached by the participants.

#### 2.6.3. Muscle Activation

Raw EMG signals recorded during MVIC and submaximal contractions were 10–400 Hz bandpass filtered, full-wave rectified, and 9 Hz low-pass filtered to obtain the linear envelope [37]. All filters were 4^th^-order, zero-lag butterworth filter. Each EMG signal was normalized to its maximal EMG value obtained when used as agonist during MVIC. The mean amplitude of the normalized EMG was averaged over the period of interest. EMG_TS_ was obtained by averaging the mean amplitude from GM, GL, and SOL. EMG_TA_ and EMG_TS_ were quantified for each trial and averaged over evaluation session independently for each torque level. 

#### 2.6.4. Corticomuscular Coherence

**Figure 2:**
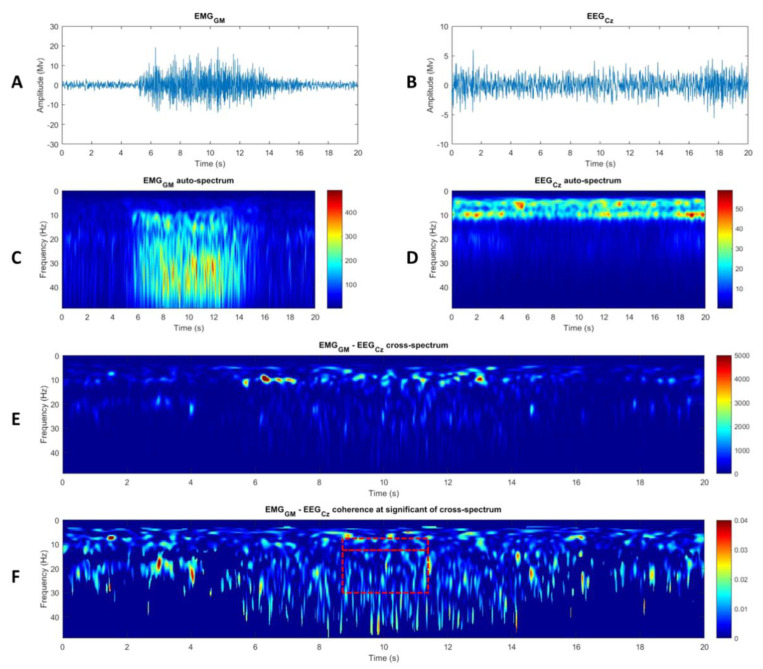
Typical recording of (**A**) Gastrocnémius Medialis (GM) electromyographic (EMG) and (**B**) Cz electroencephalographic (EEG) activities obtained during the experimental procedure. Wavelet auto-spectra of the GM EMG (**C**) and Cz EEG (**D**) signals. (**E**) Wavelet cross-spectrum and (**F**) wavelet-magnitude squared coherence between GM EMG and Cz EEG signals in the time-frequency domain. The red rectangles delimit the α (8–13 Hz) and ß (13–31 Hz) frequency band over the period of interest.

After appropriate preprocessing, the auto-spectrum of each EMG (Figure 2A,C) and Cz EEG (Figure 2D) signal and the cross-spectrum between each EMG and Cz EEG signal (Figure 2E) were quantified in the time–frequency domain using the WavCrossSpec Matlab toolbox for wavelet coherence analysis [38,39]. Wave number was set at 7 with frequency ranging from 0.05 Hz to 48.61 Hz in 0.45 Hz step. The magnitude of the CMC between Cz EEG and each EMG signal was quantified using the following equation:

(3) $$\begin{array}{ccc} Coh_{c1,c2}2\left(\omega ,u\right) & = & \frac{\left|S_{c1c2}\left(\omega ,u\right)\right|2}{\begin{array}{ccc} S_{c1c1}\left(\omega ,u\right) & \cdot & S_{c2c2}\left(\omega ,u\right) \end{array}} \end{array}$$

where $S_{c1c2}\left(\omega ,u\right)$ is the wavelet cross-spectrum between EMG and CZ EEG signals at frequency ω and time *u*, $S_{c1c1}\left(\omega ,u\right)$ and $S_{c2c2}\left(\omega ,u\right)$ are the wavelet auto-spectra of the EMG and CZ EEG signals, respectively. For each muscle, the magnitude of the CMC was computed over the period of interest in the α (8–13 Hz) and β (13–31 Hz) frequency bands with values set to zero where non-significant correlation in between Cz EEG and EMG signals was detected on the wavelet cross-spectrum [38]. The magnitude of the CMC for the TS was obtained by averaging CMC magnitude between Cz and GM, GL and SOL muscles (CMC_TS_) independently for each torque level.

### 2.7. Statistical Analysis

Statistical analysis was performed using JASP Computer software (JASP Team (2020). JASP (Version 0.12.2) [Computer software]). The normality of the distribution was assessed with a Shapiro–Wilk test. It revealed that the MVIC in PF, the EMG_TA_, the average net torque, AE, and the VE were not normally distributed. Therefore, an ANOVA with repeated measures on Evaluation time (PRE, CTR, MID, POST) was conducted for MVIC in DF. Equivalent non-parametric Friedman ANOVA was performed for MVIC in PF. 

A two-way repeated-measures ANOVA on Evaluation time (PRE, CTR, MID, POST) × Torque level (T0, Ti) was conducted on the normalized net torque, EMG_TS_, and the magnitude of CMC. Equivalent non-parametric Friedman ANOVA on (4 Evaluation time × 2 Torque level) was conducted independently on the EMG_TA_, AE, VE, and net torque recording over HFP. Huynh-Feldt correction for degree of freedom was used where applicable and *ε* values are reported. If a significant effect was found, non-directional paired *t*-tests (or equivalent non-parametric Conover’s post hoc tests) were used to compare each factor. Cohen’s d was reported where applicable. The significance level was set at *p* = 0.05 and Bonferonni-Dunn correction was applied where applicable.

## 3. Results

### 3.1. Torque Production during Maximal Voluntary Isometric Contractions

**Figure 3:**
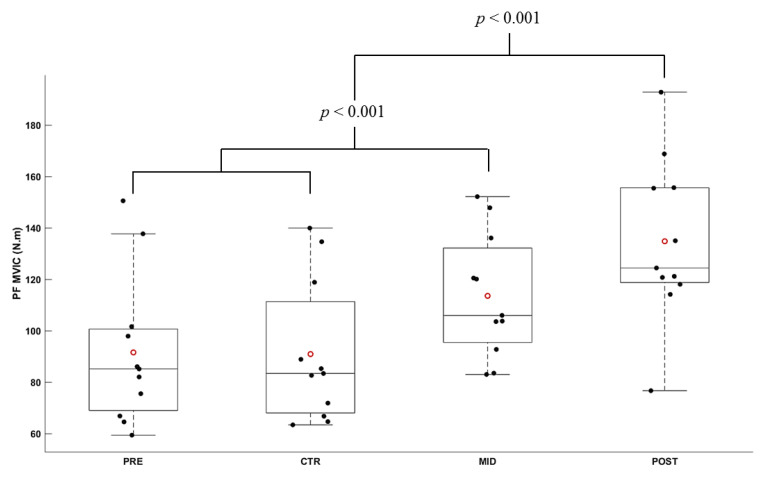
Average torque during MVIC in plantarflexion (N.m) according to the evaluation session (PRE, CTR, MID, POST sessions). Each whiskers box indicates the mean (red circle) and the median (horizontal line). The inferior edge and superior edges of the box indicate the 25th and 75th percentile, respectively. Error bars represent the most extreme and non-outlier data points. Black dots represent individual participant performance.

In plantarflexion, the Friedman ANOVA revealed a significant effect of Evaluation time on the maximal torque produced (*X*^2^ = 28.64; *df* = 3; *p* ≤ 0.001). The maximal torque produced during PRE and CTR evaluations was not significantly different (*t*_10_ = 0.433; *p* = 1.000). All other comparisons were significantly different (all |*t*_10_| < 11.691, all *p* < 0.001). Compared to PRE, the MVIC increased by 27.97 ± 10.96% for MID, and 54.01 ± 22.97% for POST evaluation. In comparison to MID, the MVIC increased by 19.03 ± 9.26% for POST evaluation. Figure 3 depicts the modulation of MVIC in PF throughout the evaluation sessions. 

No significant effect was revealed in dorsiflexion on the MVIC (*F*_3, 30_ = 0.039; *p* = 0.961; *ε* = 0.662; *η*^2^*p* = 0.004, all ‖*d*‖ ≤ 0.126). The average torque produced during MVIC in DF was 39.66 ± 2.83 N.m across all evaluation sessions. 

### 3.2. Torque Production during Submaximal Contractions

The Friedman ANOVA revealed a significant effect of Evaluation time (*X*^2^ = 31.532; *df* = 3; *p* < 0.001) and Torque level (*X*^2^ = 4.160; *df* = 1; *p* = 0.041) on the net torque. Conover’s post hoc test revealed no significant difference (all |*t*_10_| ≥ 0.628, *p* ≥ 0.724) in between submaximal torque computed over PRE and CTR evaluations and over MID and POST evaluations. All other comparisons were significantly different (|*t*_10_| ≥ 4.240, *p* < 0.001). The torque level performed at 20% of T_0_ averaged 17.79 ± 3.26 N.m, 18.04 ± 3.34 N.m, 18.17 ± 3.38 N.m, and 18.29 ± 3.40 N.m, while it increased at Ti from 17.96 ± 3.34 N.m, 17.93 ± 3.16 N.m, to 22.57 ± 2.90 N.m and 26.74 ± 3.67 N.m, respectively for PRE, CTR, MID, and POST evaluation.

**Figure 4:**
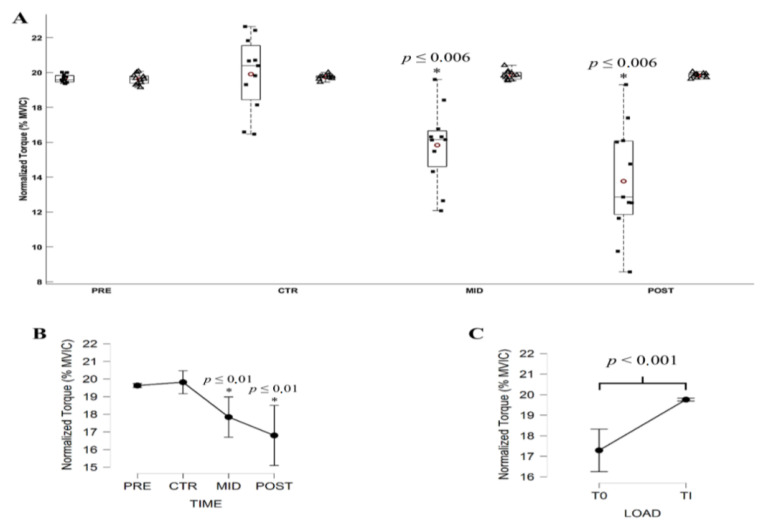
(**A**) Average normalized torque (% MVIC) during submaximal contractions computed according to the torque levels, T_0_ (black square) and Ti (white triangle), and evaluation sessions (PRE, CTR, MID, POST sessions). Each whiskers box indicates the mean (red circle) and the median (horizontal line). The inferior and superior edges of the box indicate the 25th and 75th percentile, respectively. Black dots represent individual participant performance. (**B**) Average normalized torque (N.m) during submaximal contractions computed according to evaluation sessions and (**C**) to the torque levels. Error bars represent the most extreme and non-outlier data points (**A**) and 95% confidence intervals (**B**,**C**). * significantly different from all other conditions.

The ANOVA revealed a significant effect of Evaluation time (*F*_3,30_ = 36.436; *p* <0.001; *ε* = 0.791; *η*^2^*p* = 0.785), Torque level (*F*_3,30_ = 23.773; *p* <0.001; *η*^2^*p* = 0.704) and an interaction of Evaluation time *x* Torque level (*F*_3,30_ = 36.436; *p* < 0.001; *ε* = 0.791; *η*^2^*p* = 0.785) on the percentage torque. Post hoc comparisons revealed no significant difference between the different percentage torque at Ti. Percentage torque at T_0_ computed over MID and POST were significantly different to all other conditions |*t*_10_| ≥ 3.930, all *p* ≤ 0.006). Figure 4 shows the overall modulation of the percentage torque throughout the submaximal contractions of the evaluation sessions. 

### 3.3. Accuracy and Variability of the Torque Production during Submaximal Contraction

The Friedman ANOVA revealed a significant effect of Evaluation time (*X*^2^ = 28.299; *df* = 3; *p* < 0.001) on the AE. Conover’s post hoc test revealed no significant difference (|*t*_10_| = 0.531, *p* = 1.000) in between the AE computed during the CTR and MID evaluation session. All other comparisons were significantly different (all |*t*_10_| ≥ 0.531, all *p* ≤ 0.02). Averaged AE across the two torque levels decreased from 0.69 ± 0.12%, 0.52 ± 0.07%, 0.51 ± 0.09% to 0.43 ± 0.08%, respectively, for PRE, CTR, MID, and POST evaluations. 

**Figure 5:**
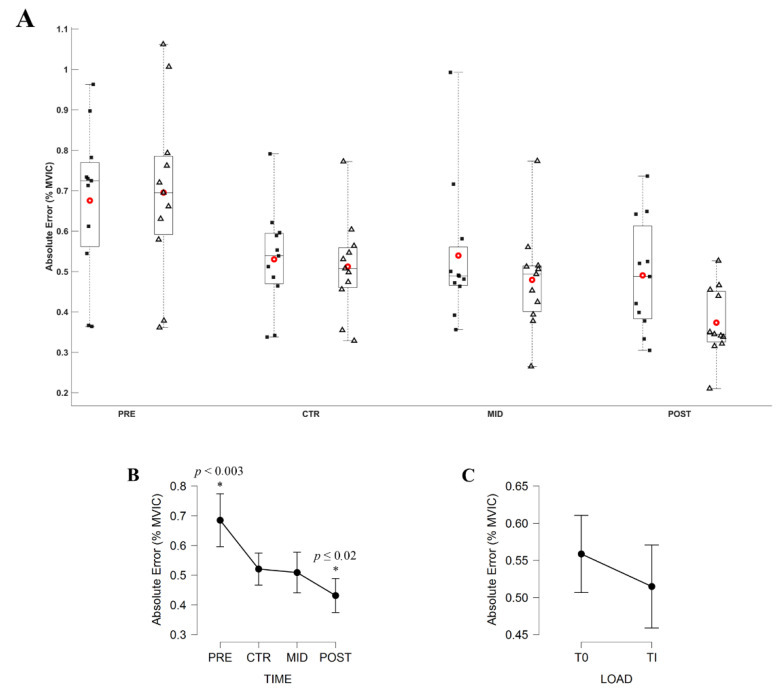
(**A**) Absolute Error (AE; % MVIC) during submaximal contractions computed according to the torque levels, T_0_ (black square) and Ti (white triangle), and evaluation sessions (PRE, CTR, MID, POST sessions). Each whiskers box indicates the mean (red circle) and the median (horizontal line). The inferior and superior edges of the box indicate the 25th and 75th percentile, respectively. Black dots represent individual participant performance. (**B**) AE during submaximal contractions computed according to evaluation sessions and (**C**) to the torque levels. Error bars represent the most extreme and non-outlier data points (**A**) and 95% confidence intervals (**B**,**C**). * significantly different from all other conditions.

**Figure 6:**
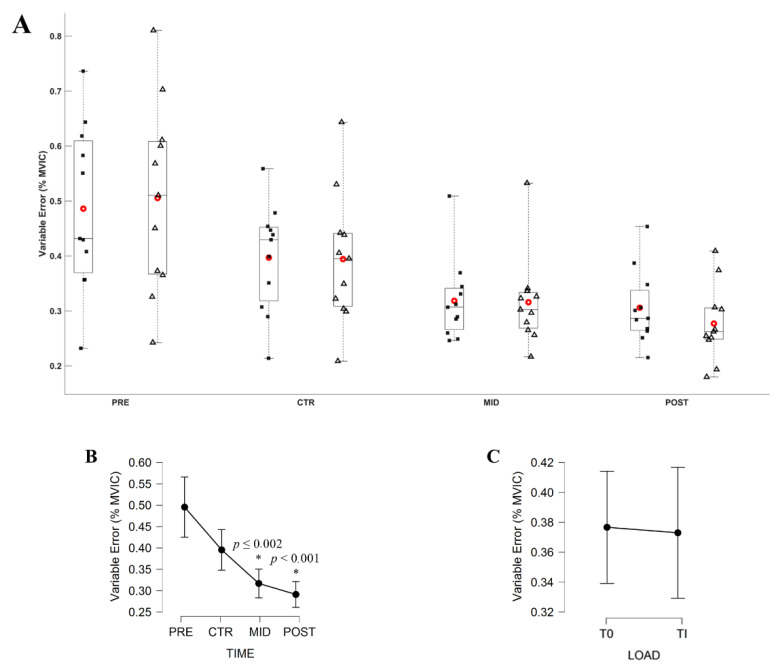
(**A**) Variable Error (VE; % MVIC) during submaximal contractions computed according to the torque levels, T_0_ (black square) and Ti (white triangle), and evaluation sessions (PRE, CTR, MID, POST sessions). Each whiskers box indicates the mean (red circle) and the median (horizontal line). The inferior and superior edges of the box indicate the 25th and 75th percentile, respectively. Black dots represent individual participant performance. (**B**) VE during submaximal contractions computed according to evaluation sessions and (**C**) to the torque levels. Error bars represent the most extreme and non-outlier data points (**A**) and 95% confidence intervals (**B**,**C**). * Significantly different from PRE and CTR evaluation.

The Friedman ANOVA revealed a significant effect of Evaluation time on the VE (*X*^2^ = 36.818; *df* = 3; *p* < 0.001). Conover’s post hoc test revealed no significant difference in between the VE computed during PRE and CTR (|*t*_10_| = 2.255, *p* = 0.163) and in between MID and POST (|*t*_10_| = 1.921, *p* = 0.352) evaluations. All other comparisons were significantly different (all |*t*_10_| ≥ 3.840, all *p* ≤ 0.002). Averaged VE across the two torque levels decreased from 0.50 ± 0.09%, 0.40 ± 0.06%, 0.32 ± 0.04%, to 0.29 ± 0.04%, respectively, for PRE, CTR, MID and POST evaluation. Figure 5 and Figure 6 respectively depict the modulation of the AE and VE throughout the evaluations. 

### 3.4. Muscle Activation

The ANOVA revealed a significant effect of Torque level (*F*_3,30_ = 10.745; *p* = 0.008; *η*^2^*p* = 0.518) and an interaction of Torque level x Evaluation time (*F*_3,30_ = 18.586; *p* < 0.001; *ε* = 0.788; *η*^2^*p* = 0.650) on the EMG_TS_. At T_0_, post hoc test revealed that EMG_TS_ decreased at POST evaluation in comparison to PRE evaluation (*F*_3,30_ = 5.616, *p* = 0.004). EMG_TS_ was lower at T_0_ in comparison to Ti at MID and POST evaluations (all ‖*t*_10_‖ ≥ 3.785, all *p* ranging from <0.001 to 0.028). 

**Figure 7:**
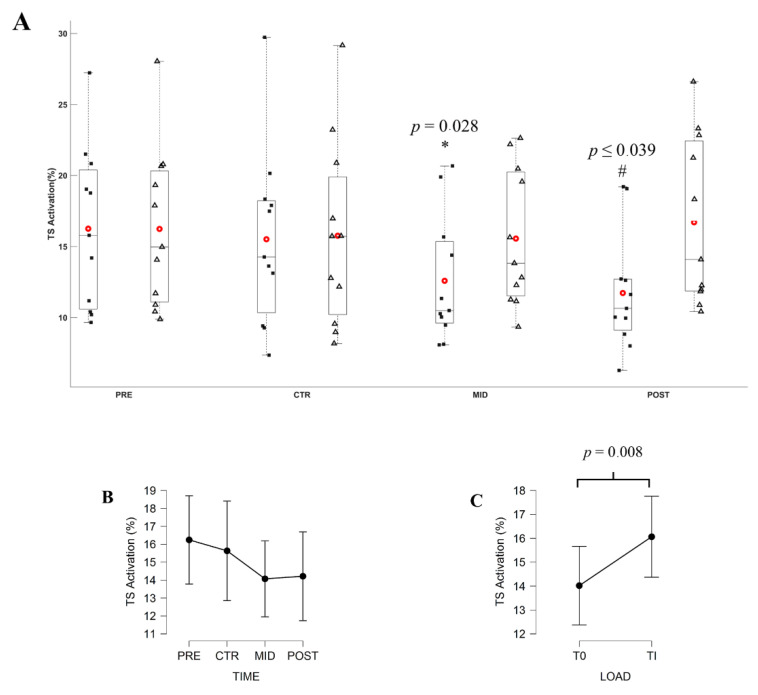
(**A**) Average EMG_TS_ during submaximal contractions computed according to the torque levels, T_0_ (black square) and Ti (white triangle), and evaluation sessions (PRE, CTR, MID, POST sessions). Each whiskers box indicates the mean (red circle) and the median (horizontal line). The inferior and superior edges of the box indicate the 25th and 75th percentile, respectively. Black dots represent individual participant performance. (**B**) Average EMG_TS_ during submaximal contractions computed according to evaluation sessions and (**C**) to the torque levels. Error bars represent the most extreme and non-outlier data points (**A**) and 95% confidence intervals (**B**,**C**). * Significant difference in comparison to Ti torque level recording at MID evaluation. # significant difference in comparison to T_0_ and Ti torque level respectively recorded at PRE and POST evaluation.

**Figure 8:**
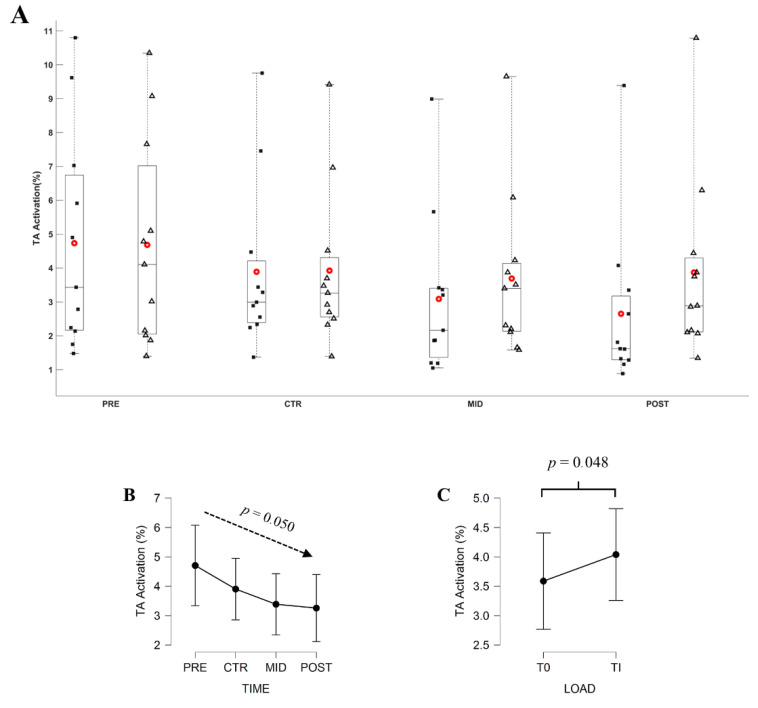
(**A**) Average EMG_TA_ during submaximal contractions computed according to the torque levels, T_0_ (black square) and Ti (white triangle), and evaluation sessions (PRE, CTR, MID, POST sessions). Each whiskers box indicates the mean (red circle) and the median (horizontal line). The inferior and superior edges of the box indicate the 25th and 75th percentile, respectively. Black dots represent individual participant performance. (**B**) Average EMG_TA_ during submaximal contractions computed according to evaluation sessions and (**C**) to the torque levels. Error bars represent the most extreme and non-outlier data points (**A**) and 95% confidence intervals (**B**,**C**).

The Friedman ANOVA revealed a significant effect of Evaluation time (*X*^2^ = 7.831; *df* = 3; *p* = 0.050) and Torque level (*X*^2^= 3.896; *df* = 1; *p* = 0.048) on the EMG_TA_. Connover’s post hoc test revealed no significant difference in between evaluations (all |*t*_10_| ranging from 0.001 to 2.483, *p* ≥ 0.092). EMG_TA_ computed over T_0_ decreased from 4.73 ± 1.92%, 3.89 ± 1.48%, 3.08 ± 1.41%, to 2.65 ± 1.44%, respectively for PRE, CTR, MID, and POST evaluations. EMG_TA_ at Ti averaged 4.68 ± 1.83%, 3.92 ± 1.37%, 3.69 ± 1.41%, and 3.87 ± 1.58%, respectively, for PRE, CTR, MID, and POST evaluations. Figure 7 and Figure 8 depict, respectively, the EMG_TS_ and EMG_TA_ throughout evaluations.

### 3.5. CMC Magnitude 

All participants exhibited significant CMC across all the evaluation sessions. 

**Figure 9:**
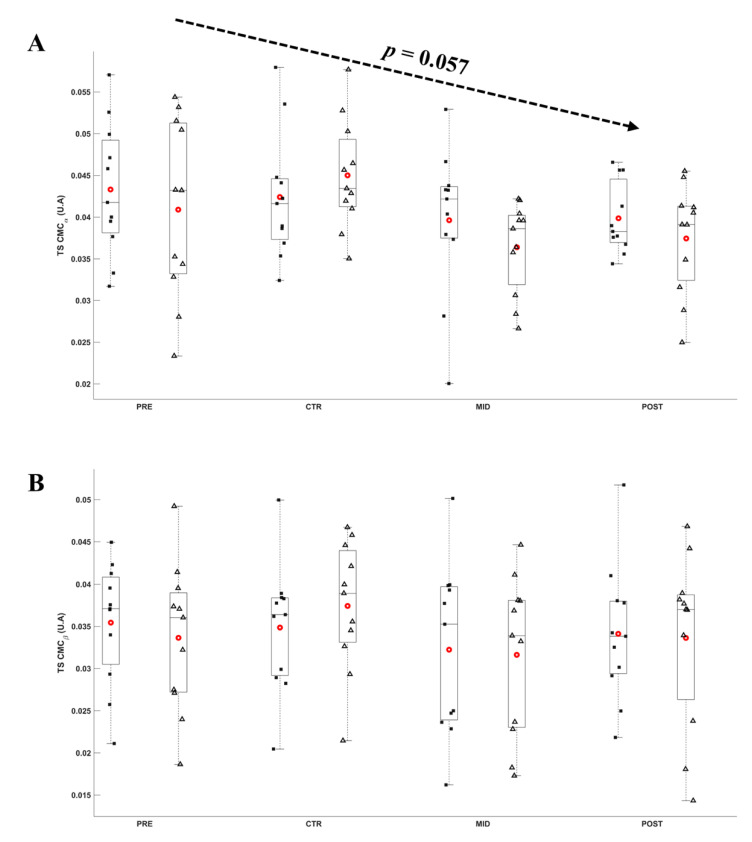
Average α (**A**) and ß (**B**) CMC_TS_ during submaximal contractions computed according to the torque levels, T_0_ (black square) and Ti (white triangle), and evaluation sessions (PRE, CTR, MID, POST sessions). Each whiskers box indicates the mean (red circle) and the median (horizontal line). The inferior and superior edges of the box indicate the 25th and 75th percentile, respectively. Black dots represent individual participant performance. Error bars represent the most extreme and non-outlier data points.

**Figure 10:**
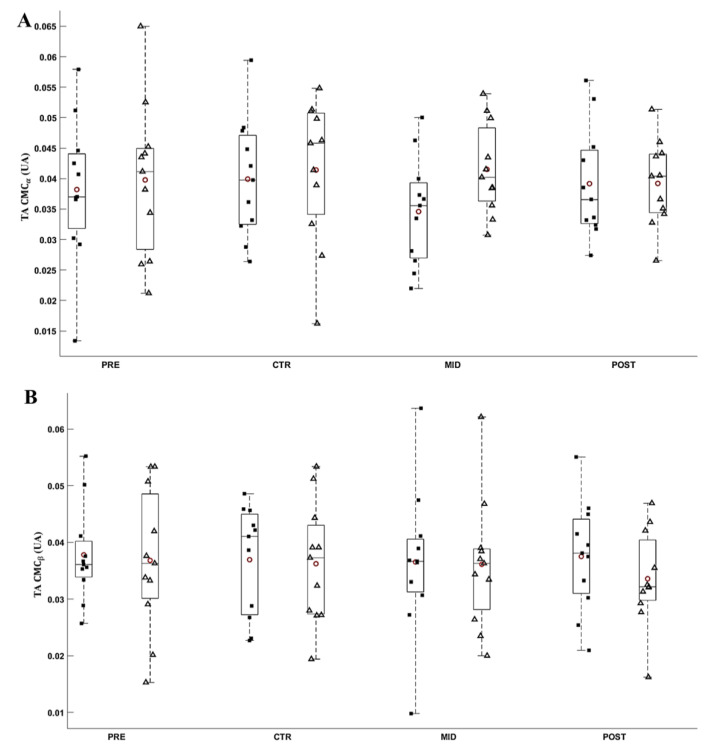
Average α (**A**) and ß (**B**) CMC_TA_ during submaximal contractions computed according to the torque levels, T_0_ (black square) and Ti (white triangle), and evaluation sessions (PRE, CTR, MID, POST sessions). Each whiskers box indicates the mean (red circle) and the median (horizontal line). The inferior and superior edges of the box indicate the 25th and 75th percentile, respectively. Black dots represent individual participant performance. Error bars represent the most extreme and non-outlier data points.

The ANOVA did not reveal any significant effect of Evaluation time (*F*_8,80_ ≥ 0.111; *p* ≥ 0.057; *ε* ≥ 0.623; *η*^2^*p* ≥ 0.011; *d* ≤ 0.93) and Torque level (*F*_8,80_ ≥ 0.018; *p* ≥ 0.156; *ε* = 1.000; *η*^2^*p* ≥ 0.002; *d* ≤ 0.46) on the CMC_TS_ and CMC_TA_. On average, CMC_TA_ was 0.039 ± 0.002 A.U. and 0.036 ± 0.002 A.U., and CMC_TS_ was 0.041 ± 0.002 A.U. and 0.034 ± 0.002 A.U., respectively, for α and ß bands across all torque level and evaluations. Figure 9 and Figure 10, respectively, depict the CMC_TS_ and CMC_TA_ throughout evaluations.

## 4. Discussion

This study evaluated the strength and corticomuscular adaptations induced by a 3 week MST specifically designed to strengthen ankle plantarflexion. Overall, the results revealed that the maximal torque produced in plantarflexion significantly increased over time, while no torque increase was shown in dorsiflexion, i.e., in the opposite “movement direction”. This result demonstrates that our MST was very specific to the trained condition. During submaximal contractions, the error and the variability of the torque production and muscle activities were decreased throughout the training. However, no CMC change was evidenced over time. These results are discussed in the subsequent sections.

### 4.1. Muscle and Torque Adaptations Are Noticeable as Soon as One Week of MST

Maximal torque produced significantly increased throughout the training in plantarflexion only. This result corroborates previous findings showing MST efficiency to quickly increase strength production [25,26,40] and extend the ST specificity to the movement direction [41,42] and task performed [43] to MST. Interestingly, at MID evaluation, maximal torque was already significantly increased in comparison to the PRE evaluation. To our knowledge, no study assessed the strength increase as soon as the first week of MST. It is unlikely that this result is related to the discovery of the experimental task since no torque difference was revealed between the PRE and CTR evaluations. Previous studies using electrostimulation techniques demonstrated that the strength increases observed after MST are mainly driven by corticospinal factors. The authors especially proposed that the observed effects reflect an increase in the transmission of the descending inputs to the spinal motoneurons, leading to an increase of motor units recruitment or higher motor units firing frequency [24,44]. In the current study, the relatively short MST period allows us to assume that the increase in strength production should reflect neurophysiological adaptations rather than muscle mass adaptations [18]. Similar strength increases attributable to ST ranging from 44% to 54% were previously reported following four training weeks [45,46]. Although structural muscle adaptations to isometric ST may take up to 3 months to occur [47,48], Achilles tendinous adaptations have been evidenced following short ST intervention [49]. Therefore, the large strength increase observed in the current study could be attributed to structural adaptations of the tendon structures and to the neural adaptation of the motor commands. 

Indeed, some neural adaptations are noticeable during submaximal contractions. The EMG_TS_ significantly decreased over time, concurrently with the percentage torque decrease at initial torque level (i.e., similar torque production), while it remained constant when contractions were performed at constant percentage torque level (i.e., increased torque production throughout evaluation). As suggested by a previous study [3,4,6], the EMG_TA_ decreased throughout the MST completion for both initial and daily torque level. Besides, the EMG_TA_ activation was significantly different between the initial and daily torque levels as the training progressed. This latter result is corroborated by many studies which evidenced the increase of the antagonist activation as the torque level increases [13,30,50]. These results suggest a decrease in the relative effort to reach the initial torque level and no change in the relative effort to reach the daily torque level. To our knowledge, no study investigated the level of effort to reach constant torque representing the initial level of performance. However, the previous findings reported the effectiveness of MST to enhance work economy [22], mechanical efficiency [26], and walking performance [27], which represent submaximal torque intensity during daily activities. These results highlight the effectiveness of MST to decrease the level of effort to reach a constant submaximal torque level, and the associated decreased in antagonist activity.

### 4.2. MST Improves Motor Control of Submaximal Contractions

As a consequence of MVC torque increase, the difference between the initial and daily submaximal torque levels significantly increased throughout the experimental procedure. This difference was highlighted with the torque increase at submaximal daily torque level and percentage torque decrease at submaximal initial torque level.

As expected, the AE and the VE were decreased throughout the experimental procedure at both daily and initial torque levels. Although the training procedure had not started, the AE decreased in between PRE and CTR evaluations. Noteworthy is that both EMG_TS_ and EMG_TA_ remained unaltered in between PRE and CTR evaluations, suggesting that the increased accuracy in CTR evaluation may be a consequence of discovering the experimental procedure. The AE remained unaltered at the MID evaluation session compared to CTR, then decreased again at POST evaluation. These results suggest that, after initial adaptation to the experimental procedure, the adaptation of the torque accuracy induced by the MST may take time to be fully efficient, as results only show significant improvements after three weeks. Previous findings showed that the ST procedure induced increased force accuracy at submaximal force level [5,28]. These results suggested that increased force accuracy could be transferred over a daily task executed at a low submaximal force level. However, the trainings lasted 6 and 10 weeks, and no evaluation sessions were performed during the training. The current study brings additional information about the acute changes in the torque production accuracy after ST initiation and extends these results into MST, although part of these changes could be attributable to the discovery of the experimental task. 

The VE computed over the period of interest only decreased after the first week of MST. According to previous findings, the force variability should have continuously decreased over time as soon as one week [21] and throughout the ST initiation [29]. After the completion of a ST, Tracy et al., 2004, reported additional decrease in the force variability [29]. However, their training lasted 4 weeks, i.e., one week longer than the current MST, which could explain the constant VE after the MST completion. These results could highlight the acute effectiveness of the MST to increase the motor control efficiency by enhancing both accuracy and variability of the torque production over different submaximal torque levels. 

### 4.3. CMC Is Not Altered after 4 Week MST

In contradiction with our hypothesis, our MST procedure failed to alter the CMC magnitude, despite the torque and muscle adaptations. Studies investigating effects of ST on CMC magnitude reported decreased ß-band CMC magnitude in expert population accounting for, at least, 3 years of practice [7,11]. Other studies reported significant short-term effects of visuomotor training, increasing the α-band and ß-band CMC magnitude [17,20], suggesting a relationship in between the CMC magnitude and fine motor control task. However, the performed experimental and training tasks largely differ from the strength training procedure. These early increases of CMC magnitude could reflect a higher cortical involvement to better perform the motor task. Our results could indicate, on the one hand, that despite the large torque and muscle adaptations induced by the MST procedure, the long-term neurophysiological adaptations may need more time to be quantified with a decrease of the CMC magnitude or, on the other hand, that the current experimental procedure did not sufficiently involve fine motor control to increase the CMC magnitude. Interestingly, some studies demonstrated that the reproducibility of the coherence magnitude values is highly variable, ranging from poor to excellent [51,52,53], and large absolute changes appear to be required to indicate a real difference. In our study, the CMC in the α-band presented a tendency to decrease (*p* = 0.057) throughout the training intervention, in association to a large size effect (*d* ≤ 0.93). This result allows us to hypothesize that a longer training duration could be relevant to significantly decrease the CMC magnitude in both α- and ß-band, as reported by previous studies [7,11]. Finally, recent findings suggest that the muscle composing the TS could share less common input in comparison to other muscle groups [54]. The authors suggest that this strategy may allow more flexible control to comply both movement execution and balance control. Whether the TS muscles require more flexible control associated to less common input could explain the absence of training effect of the modulation of the CMC magnitude throughout the current study.

## 5. Conclusions

This study proposed an original and adaptive maximal-strength training to quickly increase ankle strength production. The training intensity was adjusted day by day using the daily MVIC. Results showed acute torque increase only in plantarflexion without change in dorsiflexion, suggesting specific adaptations induced by short-term MST. Objective markers of the motor control as VE and AE significantly decreased over time. The relative level of effort to reach initial torque level was decreased as well as antagonist muscle activation for initial and daily torque level. Nevertheless, although both significant agonist and antagonist CMC were reported, no modulation of the CMC magnitude with training was revealed. Further investigations are needed to investigate the long-term neurophysiological adaptations changes induced by such MVIC-based MST. As MST has been previously shown to enhance strength and functional performance in stroke population [40,55], similar experiments need to be carried out in a clinical environment to evaluate the relevance of MVIC-based MST into clinical environment to quickly improve strength production and motor control efficiency.

## Data Availability

The data are not publicly available because of ethics committee restriction.

## Supplementary Materials

The following are available online at https://www.mdpi.com/2076-3425/11/2/254/s1, supplementary materials: Quantification of training accuracy.
