# Supplementary material for: Corticomuscular Coherence and Motor Control Adaptations after Isometric Maximal Strength Training
Source: Brain Sci. 2021 Feb 18;11(2):254. doi: 10.3390/brainsci11020254 (PMC7922221; doi:10.3390/brainsci11020254)
Supplement: Supplementary file 1 [file brainsci-11-00254-s001.pdf]

Supplementary Materials: Quantification of training accuracy

1. Data Processing

The net torque recorded during training sessions was 10-Hz low-pass filtered using $\mathbf{4}^{th}$ order, zero lag butterworth filter [34]. The MVIC was defined as the highest average of a 2-s sliding window ran over the entire period of contraction. Modulation of training MVICs were respectively expressed as percentage of the first training session (T01).

Two training characteristics were quantified to objectively control the accuracy of the training: Effective Time over 85% of MVIC (ET) and Relative Training Intensity (RTI). The ET was defined as the time spent above 85% MVIC, *i.e.*, the lowest threshold of the training level. The ET was quantified for each contraction and averaged over training session. The RTI was defined as the highest average torque of a 2-s sliding window ran over the entire period of contraction normalized by daily MVIC. The RTI was quantified for each contraction and averaged over training session.

2. Statistical Analysis

Statistical analysis was performed using JASP Computer software (JASP Version 0.10.0; 2018). Normality of the distribution was assessed with Shapiro-Wilk test. MVIC were normally distributed ($p \geq 0.082$). A repeated measures ANOVA (T01, T02, T03, T04, T05, T06, T07, T08, T09) on *Training time* was conducted on the training MVIC. ET and RTI data were not normally distributed (all $p < 0.001$ to 0.975). A non-parametric Friedman ANOVA with repeated measures on *Training time* was conducted on the ET and the RTI. If a significant effect was found, non-directional paired *t*-tests (or equivalent non-parametric Conover's post hoc tests) were used to compare each factor and Cohen's d was reported where applicable.

3. Results

The ANOVA revealed a significant effect of *Training time* on the MVIC produced in PF ($F_{8,80} = 13.34$; $p < 0.001$; $\varepsilon = 0.574$; $\eta^2 p = 0.572$, all $||d||$ ranging from 0.140 to 2.367). The figure A1 shows the overall modulations of the MVIC in PF throughout the training sessions. Significant modulations of the MVIC revealed by Conover post-hoc tests are summarized into the table A1 as a percentage of change between the different training sessions.

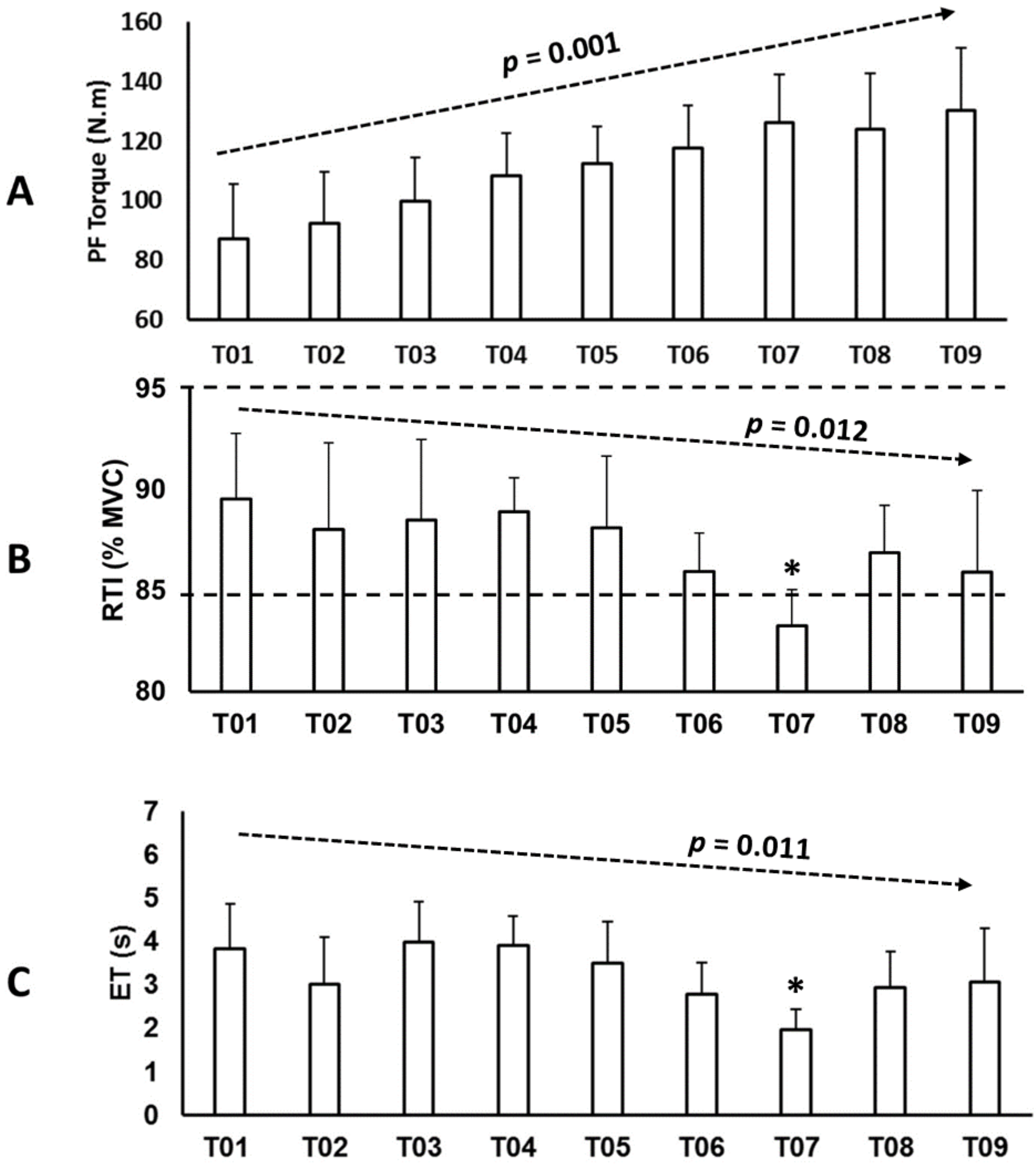

Figure A1: (A) Average torque (N.m) during MVIC in PF, (B) Relative Training Intensity (RTI; % MVIC), and (C) Effective Time (ET; s). All variables were recorded during the nine training sessions and include all participants. Error bars represent 95% confidence interval. Dashed lines in B represent the thresholds of training level (90±5%) and * indicates significant torque difference in comparison from T01 to T05 training sessions.

The Friedman ANOVA revealed a significant effect of *Training time* on the RTI ($X^2 = 19.59$; df = 8; $p = 0.012$). Conover's post hoc test revealed that T07 was significantly different than T01 to T05 (all p ranging from 0.001 to 0.014). Except for T07 (83.24 ± 1.81 %), RTI was over than 85% for all training sessions (*fig.* A2). Mean RTI was 87.23 ± 1.28 % including all trials. The Friedman ANOVA revealed a significant effect of *Training time* on the ET ($X^2= 19.81$; df = 8; $p = 0.011$). Conover's post hoc test revealed that T07 was significantly different than T01 to T05 ($p \leq 0.001$). Averaged ET was 3.22 ± 0.33 s including all training sessions (*fig* 1.C). Table A2 summarizes the RTI and ET modulation throughout the training procedure.

Table 1: Average (± 95% confidence interval) percentage changes comparison in plantarflexion torque during training sessions.

	T01	T02	T03	T04	T05	T06	T07	T08
**T02**	8.32 ±11.14							
**T03**	19.59 ±13.92	13.27 ±18.63						
**T04**	30.45 ±15.08	23.77 ±19.78	10.18 ±10.28					
**T05**	38.15 ±19.59	30.51 ±25.56	14.76 ±8.33	5.65 ±9.74				
**T06**	42.49 ±17.60	33.32 ±18.41	19.17 ±7.36 *	10.60 ±12.63	4.85 ±8.05			
**T07**	52.27 ±16.30 *	43.38 ±19.78 *	28.37 ±9.77 *	17.73 ±8.36 *	13.04 ±10.13	8.73 ±10.65		
**T08**	48.10 ±15.72 *	37.76 ±13.86 *	26.33 ±13.70 *	16.03 ±13.52	11.72 ±13.70	6.40 ±11.78	-1.75 ±8.07	
**T09**	54.61 ±15.93 *	45.03 ±16.68 *	30.99 ±9.57 *	20.75 ±11.50	16.06 ±12.64	10.77 ±10.06	2.31 ±5.16	5.63 ±8.02

**** indicates significant torque difference in between training sessions.***

Table A2: *Average (± 95% confidence interval) Relative Training Intensity (RTI, % MVIC) and Effective Time (ET, s) computed throughout the training procedure.*

	T01	T02	T03	T04	T05	T06	T07	T08	T09
**RTI (% MVIC)**	89.53 ± 3.26	88.03 ± 4.30	88.48 ± 4.00	88.91 ± 1.67	88.11 ± 3.56	85.94 ± 1.90	83.24 ± 1.81*	86.88 ± 2.35	85.90 ± 4.05
**ET (S)**	3.84 ± 1.03	3.02 ± 1.08	3.98 ± 0.93	3.92 ± 0.67	3.51 ± 0.96	2.80 ± 0.73	1.97 ± 0.45*	2.95 ± 0.83	3.08 ± 1.23

**** indicates significant torque difference in comparison from T01 to T05 training sessions.***

4. Discussion

The current training included a day-by-day adjustment of the training intensity according to the daily MVIC. MVIC is a fast and reliable procedure [56] where one participant has to develop as quickly and strongly as possible the maximum joint torque against a dynamometer device [57, 58]. It requires as few as 3 trials [10] and do not lead to excessive fatigue allowing to consider daily maximal strength production evaluation to adjust training intensity. Although several studies investigated regular strength training based on MVIC and demonstrated significant torque improvement in healthy people [22, 57, 59-61] and cerebral palsy patients [62], no study strictly investigated MVIC-based MST. Overall,

our results revealed that the torque produced in plantarflexion significantly increased over time while the training performance remained unaltered.

The design of our MVIC-based MST brought additional information regarding the training completion through the evaluation of daily MVIC, RTI and ET. To our knowledge, only one previous study [63] aimed to objectively quantify the training completion of a MVIC-based training. In this study, the authors evaluated the number of maximal contractions successfully maintained more than 3s during the training. Unfortunately, the authors did not evaluate the absolute torque intensity nor the relative training intensity reached by participants, which may be variable during the training. The present study proposed RTI and ET as two objective markers to assess the training accuracy. As expected, while torque production increased throughout the training procedure, the day-by-day adjustment of the training intensity did not remarkably alter RTI and ET, suggesting no training changes for the participants when attempting to maintain the lower threshold intensity. However, during the three weeks of training, the seventh training session revealed a significant decrease of ET and RTI. These results should underline the participants' difficulties to reach and maintain the threshold level at the beginning of the third training week while the PF-MVIC continuously increased. Indeed, although non-significant, a first decrease of the daily MVIC was also noted at the eighth training session in comparison to the seventh training session. However, the RTI and ET reached during the eighth training went back up to the expected levels. Therefore, RTI and ET could be good markers of fatigue and may be used to adjust the recovering period following the training. In practice, the advent of low-cost transportable dynamometer could expand MVIC-based trainings performed in both laboratory and home environment [63].

References (not included in the main text):

56. Suchomel, T. J., Nimphius, S., & Stone, M. H. (2016). The Importance of Muscular Strength in Athletic Performance. *Sports Medicine*, *46*(10), 1419‑1449.
57. Lee, S. E. K., Lira, C. A. B. de, Nouailhetas, V. L. A., Vancini, R. L., & Andrade, M. S. (2018). Do isometric, isotonic and/or isokinetic strength trainings produce different strength outcomes? *Journal of Bodywork and Movement Therapies*, *22*(2), 430‑437.
58. Vila-Chã, C., & Falla, D. (2016). Strength training, but not endurance training, reduces motor unit discharge rate variability. *Journal of Electromyography and Kinesiology*, *26*, 88‑93.
59. Dragert, K., & Zehr, E. P. (2011). Bilateral neuromuscular plasticity from unilateral training of the ankle dorsiflexors. *Experimental Brain Research*, *208*(2), 217‑227.
60. Selkowitz, D. M. (1985). Improvement in isometric strength of the quadriceps femoris muscle after training with electrical stimulation. *Phys. Ther*, 186–196.
61. Sidaway, B., & Trzaska, A. (Robinson). (2005). Can Mental Practice Increase Ankle Dorsiflexor Torque? *Physical Therapy*, *85*(10), 1053‑1060.
62. Stackhouse, S. K., Binder-Macleod, S. A., Stackhouse, C. A., McCarthy, J. J., Prosser, L. A., & Lee, S. C. K. (2007). Neuromuscular Electrical Stimulation Versus Volitional Isometric Strength Training in Children With Spastic Diplegic Cerebral Palsy : A Preliminary Study. *Neurorehabilitation and Neural Repair*, *21*(6), 475‑485.
63. Sun, Y., Ledwell, N. M. H., Boyd, L. A., & Zehr, E. P. (2018). Unilateral wrist extension training after stroke improves strength and neural plasticity in both arms. *Experimental Brain Research*, *236*(7), 2009‑2021.
